# The intuitive use of laryngeal airway tools by first year medical students

**DOI:** 10.1186/1471-227X-9-18

**Published:** 2009-09-22

**Authors:** Johannes Bickenbach, Gereon Schälte, Stefan Beckers, Michael Fries, Matthias Derwall, Rolf Rossaint

**Affiliations:** 1 Department of Surgical Intensive Care, University Hospital RWTH Aachen, Germany; 2 Department of Anesthesiology, University Hospital RWTH Aachen, Germany

## Abstract

**Background:**

Providing a secured airway is of paramount importance in cardiopulmonary resuscitation. Although intubating the trachea is yet seen as gold standard, this technique is still reserved to experienced healthcare professionals. Compared to bag-valve facemask ventilation, however, the insertion of a laryngeal mask airway offers the opportunity to ventilate the patient effectively and can also be placed easily by lay responders. Obviously, it might be inserted without detailed background knowledge.

The purpose of the study was to investigate the intuitive use of airway devices by first-year medical students as well as the effect of a simple, but well-directed training programme. Retention of skills was re-evaluated six months thereafter.

**Methods:**

The insertion of a LMA-Classic and a LMA-Fastrach performed by inexperienced medical students was compared in an airway model. The improvement on their performance after a training programme of overall two hours was examined afterwards.

**Results:**

Prior to any instruction, mean time to correct placement was 55.5 ± 29.6 s for the LMA-Classic and 38.1 ± 24.9 s for the LMA-Fastrach. Following training, time to correct placement decreased significantly with 22.9 ± 13.5 s for the LMA-Classic and 22.9 ± 19.0 s for the LMA-Fastrach, respectively (p < 0.05). After six months, the results are comparable prior (55.6 ± 29.9 vs 43.1 ± 34.7 s) and after a further training period (23.5 ± 13.2 vs 26.6 ± 21.6, p < 0.05).

**Conclusion:**

Untrained laypersons are able to use different airway devices in a manikin and may therefore provide a secured airway even without having any detailed background knowledge about the tool. Minimal theoretical instruction and practical skill training can improve their performance significantly. However, refreshment of knowledge seems justified after six months.

## Background

Mortality of "sudden cardiac death" (SCD) in Europe runs up to 375.000 patients per year [1] and is in most cases caused by acute (cardiac failure) coronary syndromes. To prevent secondary hypoxic damage to the brain and other vital organs due to respiratory failure, it is of paramount importance to assess and control the airway.

Several devices have been recommended helping to keep the airway open [2]. While still bringing out the "gold-standard" with the tracheal tube, it has already been shown before that the laryngeal mask airway (LMA) and the Combitube are possible alternative tools. In comparison to bag-valve facemask ventilation, they may firstly reduce the risk of gastric regurgitation and pulmonary aspiration and secondly allow more effective ventilation on the other hand [3-5].

In case of emergency, first responders mostly belong to non-physician personnel. Therefore particular training programmes should be held to meet the requirements of this specific target group. It is essential to teach and train basic life support not only with mouth-to-mouth- or bag-valve-facemask-ventilation but also with integrated airway management. Because of that a training concept is supposed to be applied and evaluated on its educational quality.

Although several data has already demonstrated a safe use of different LMA by inexperienced personnel [6-8], there is no evident consensus regarding length and content of such a training concept by this time.

The insertion of a laryngeal airway might actually be taught within a simple but well-directed training concept. Detailed background knowledge about the instrument itself might not be of relevance.

The aim of the study was to investigate the intuitive use of different laryngeal airway devices by first-year medical students. Therefore, the insertion of a LMA-Classic and a LMA-Fastrach was compared in a resuscitation model. The effects of a short teaching programme and the improvement on the laypersons' performance after these minimal theoretical instructions were examined. Moreover, the retention of skills was reviewed to evaluate long-term effects.

## Methods

### Subjects; Ethical Considerations

Subjects embodied were 139 first year medical students. They were tested at the very beginning of their studies, during their first two weeks at the medical faculty of the University of Aachen. All subjects were informed that their performance would be evaluated and used for scientific purposes. No personal data was collected. Furthermore, no influence on the health of the subjects was expected. Therefore, the local research ethical committee of the RWTH Aachen waived to obtain informed consent from each person. None of the subjects were prompted or prepared in any way prior to the study.

### Equipment

The laryngeal airway devices tested were the LMA-Classic™ and the LMA-Fastrach™ (LMA Vertriebs-GmbH, Germany). Both instruments were applied in size 4. To standardize cuff inflation volume the recommended maximum was used. Via a ventilatory tube the trachea was connected with a volumeter on which the tidal volume could be read after positioning the airway tool. The exact time from first handling the device to correct insertion was recorded with a laboratory stop watch (Junghans, Germany).

The airway trainer (Laerdal, Norway) was used as a model for insertion of the two airway devices. The airway trainer was placed on a table and therefore easily accessable.

### Study protocol

After randomization, the students were assigned to insert either the LMA-Classic or the LMA-Fastrach. Three physicians skilled in providing and teaching Advanced Life Support (ALS) (certified Instructors of the European Resuscitation Council, ERC) were present during the whole performance of each student and recorded time until the particular device was meant to be placed correctly. All tested persons were instructed with the same standardized sentence: "*This patient is unconscious and not breathing. The device in front of you may help to keep the airway open. Please insert the instrument as you consider it correctly*". The test ended when the subject confirmed the correct position in his opinion. Afterwards the cuff was inflated by the observer according to the manufacturer's suggestions. Tidal volume was measured with a volumeter by ventilating with an ambu bag. A tidal volume under 150 ml was considered as insufficient.

Beside measuring the time to correct placement of the laryngeal airway, number of attempts and initial tidal volume were documented. Air leakage was identified by audible sound during ventilation. Stop criterion was no successful insertion after 180 seconds.

After having completed the test, each student was handed out a standardized questionnaire to evaluate whether they had gained experience with laryngeal airway devices prior to the study or whether they had any medical pre-education (e.g. nurse, paramedic. etc.). After a period of one week all students were re-evaluated on the same scenario using the same device. During this week they attended two ERC-Instructor-supervised lectures consisting of one hour theoretical basics referring to the need and purpose of airway management and another hour of practical training.

The very same study protocol was repeated with the same study group six months thereafter. Again each student was evaluated before and after the above mentioned training programme.

### Statistical Data Analysis

Primary end point of the study was to determine the time from beginning the scenario to correct insertion of the laryngeal airway after the students' opinion.

Normal distribution of the data was confirmed using the Kolmogorov-Smirnov-test. By use of a t-test differences in time until correct placement and initial tidal volume between the first and the second evaluation were calculated as well as between the two different devices for each time point. All data was described as mean ± SD. A p-value of ≤ 0.05 was considered to indicate significance. For analysis statistical software SPSS 14.0 (SPSS Inc., Chicago, Ill.) was used.

## Results

The mean age of the study population was 20.7 ± 2.9 years (range 18-42). In the first evaluation, 20 out of 79 subjects in the LMA-Classic-group and 11 out of 60 subjects in the LMA-Fastrach-group failed to generate an initial tidal volume greater than 150 ml. Mean time for correct placement was 55.5 ± 29.6 s for the LMA-Classic and 38.1 ± 24.9 s for the LMA-Fastrach (p < 0.05). Numbers of attempts needed were 2.0 ± 1.6 for the LMA-Classic and 1.5 ± 0.73 for the LMA-Fastrach, respectively. The measured tidal volume with the volumeter was 674 ± 133 ml for the LMA-Classic and 1057 ± 158 ml for the LMA-Fastrach.

Air leakage at the outer end of the airways was observed in 2 cases for the LMA-Classic and in 2 cases for the LMA-Fastrach, no placement was feasible in 2 cases for both devices, respectively.

In the second evaluation, initial tidal volume <150 ml for the LMA-Classic was observed in 14 out of 79 subjects and in 6 out of 60 subjects for the LMA-Fastrach. Time until correct placement decreased significantly for both devices. In detail, mean time for the LMA-Classic was 22.9 ± 13.5 s, correct placement for the LMA-Fastrach was 22.9 ± 19.0 s. Comparing LMA-Classic with the LMA-Fastrach, no significantly faster placement could be shown. Numbers of attempts until correct placement were 1.1 ± 0.52 for the LMA-Classic and 1.4 ± 0.95 for the LMA-Fastrach.

The measured tidal volume was 777 ± 367 ml for the LMA-Classic. For the LMA-Fastrach, a tidal volume of 1018 ± 50 ml was recorded. Observed leakage was in 2 cases for the LMA-Classic and in 2 cases for the LMA-Fastrach. No successful placement was recorded in 2 cases for the LMA-Classic and for the LMA-Fastrach

An overview of the results can be found in the Table 1. The results of the questionnaire containing the students' background knowledge can be seen in Table 2.

**Table 1:** Results of the first phase

	**LMA-Classic**	**LMA-Fastrach**
Number, n	79	60

Age, mean ± SD	20.4 ± 1.7	20.7 ± 2.1

Male, n (%)	16.9 (28.8)	17.0 (32.7)

**First evaluation**		

Time, s ± SD	55.5 ± 29.6^†^*	38.1 ± 24.9^†^*

Number of attempts, n, mean ± SD	2.0 ± 1.6	1.5 ± 0.73

Tidal volume ≤ 150 ml, n (%)	20 (25.3)	11 (18.3)

Tidal volume (ml), mean ± SD	674 ± 133^†^*	1057 ± 158^†^

No Leakage, n (%)	57.0 (96.7)	44.9 (86.5)

Impossible placement, n (%)	2 (3.4)	2 (3.8)

**Second evaluation**		

Time, s ± SD	22.9 ± 13.5*	22.9 ± 19.0*

Number of attempts, n, mean ± SD	1.1 ± 0.6	1.4 ± 0.8

Tidal volume ≤ 150 ml, n (%)	14 (17.7)	6 (10)

Tidal volume (ml), mean ± SD	777 ± 367^†^*	1018 ± 50^†^

No Leakage, n (%)	49.1 (83.3)	46.7 (93.8)

Impossible placement, n (%)	2 (3.4)	2 (3.8)

^† ^Significant difference between LMA-Classic and LMA-Fastrach (p < 0.05)

* Significant difference between first and second evaluation (p < 0.05)

**Table 2:** Background knowledge; testing questions

	**Number, n (%)**
**Medical education:**	
**1) Paramedic**	9 (8.1)
**2) Nurse**	7 (6.3)
**3) None**	95 (85.6)

**First aid for drivers license**^§^	82 (73.8)

**First-aid-course**	21 (18.9)

^§ ^Mandatory by government, does not include airway management

After six months, 75 subjects were embodied in the LMA-Classic, and 60 subjects were embodied in the LMA-Fastrach group.

Again, the LMA-Fastrach could be placed significantly faster when compared with the LMA-Classic (43.1 ± 34.7 vs 55.6 ± 29.9, p < 0.05). The second evaluation after the training programme yielded a significantly decreased time period until correct placement for both devices (23.5 ± 13.2 vs 26.6 ± 21.6, p < 0.05). No statistical significance could be shown between the groups. Further results are summarized in Table 3.

**Table 3:** Results of the second phase (retention of skills, after 6 months)

	**LMA-Classic**	**LMA-Fastrach**
Number, n	75	60

**First evaluation**		

Time, s ± SD	55.6 ± 29.9^†^*	43.1 ± 34.7^†^*

Number of attempts, n, mean ± SD	2.0 ± 1.5	2.0 ± 1.3

Tidal volume ≤ 150 ml, n (%)	11 (14.7)	10 (16.4)

Tidal volume (ml), mean ± SD	556 ± 160^†^*	902 ± 389^†^

No Leakage, n (%)	59 (78.7)	45 (73.8)

Impossible placement, n (%)	2 (3.4)	2 (3.8)

**Second evaluation**		

Time, s ± SD	23.5 ± 13.2*	26.6 ± 21.6*

Number of attempts, n, mean ± SD	1.1 ± 0.5	1.4 ± 0.9

Tidal volume ≤ 150 ml, n (%)	10 (13.3)	6 (9.8)

Tidal volume (ml), mean ± SD	765 ± 267^†^*	930 ± 290^†^

No Leakage, n (%)	64 (85.3)	53 (86.9)

Impossible placement, n (%)	2 (3.4)	2 (3.8)

^† ^Significant difference between LMA-Classic and LMA-Fastrach (p < 0.05)

* Significant difference between first and second evaluation (p < 0.05)

## Discussion

The aim of our study was to evaluate the intuitive use of different laryngeal airway devices. Moreover, the improvement of use after a structured educational programme was examined.

As a major finding, we could demonstrate that, prior to training, the LMA-Fastrach could be placed significantly faster compared to the LMA-Classic. In the second evaluation, a significantly faster placement and therefore an improvement in the use were recorded for both devices. Even without any instruction, the majority of the subjects intuitively used and placed both the LMA-Classic and the LMA-Fastrach correctly. Within a further evaluation after six months, comparable results were achieved.

In our study, it could be shown, that the majority of subjects was able to insert the airway instrument adequately even without having any medical education, background knowledge or detailed technical information about the device. Hence, the results are quite consistent to already existing studies [6,9].

Other studies have also investigated the efficiency of only short and simple training periods [10]. However, the effect of time on the retention of skills gained in this study is unknown and requires further investigation.

Addressing to this problem, it could be shown that retention of skills varies in different studies, especially for different devices [11,12].

Re-evaluating retention of skills after six months, comparable results could be shown concerning time to placement, number of attempts and leakage for both devices. After refreshment, once more including theoretical background and practical skill training, the students again significantly improved in both groups.

It can be stated that regular training is therefore required to retain practical skills concerning the use of LMA.

Concerning training, our programme consisted of a short theoretical instruction and a practical skill training of overall two hours. Our results reinforce not only the fact that a clear recommendation on a training programme needs to be developed, but it also underlines that a short and well-directed training concept of overall two hours might be sufficient, at least for short-term effects. It is likely true that training programmes might even be of a shorter duration. By this time we conceptionalized this course embedded into a new Medical Reform Curriculum Aachen [13]. In a problem-oriented approach to medical education, the first year medical students received defined teaching objectives concerning Basic Life Support including AED use and, as seen in this context, airway management. The extremely positive evaluation of the new approach encouraged us to further promote this concept.

To our knowledge, there is no evident data or clear existing guideline that shows a specific time frame for an airway management training programme. A definite duration of theoretical introduction with or without practical skill training for inexperienced people is not described until now. Garcia-Guasch and co-workers compared the use of LMA with a cuffed oropharyngeal airway and a face mask in a resuscitation model in inexperienced personnel [14]. However, they did not show improvement in performance or point out a time frame for training. Yet, it seems quite clear that the use of laryngeal masks improves the quality of ventilation when compared to face mask [15].

With these results, we affirm the opinion of implementing airway management into an early stage of first aid measures.

It might have been helpful to split up another "control group" of students which did not attend the training programme. Thereby, we could have examined whether or whether not they might have improved only due to redundant performance within their second evaluation even without training sessions. However, we cannot exclude the possibility that the initial use of the laryngeal airway devices has had a training effect on the performance of the students by itself. Clearly it would be beneficial to address this issue using a more refined study design, i.e. cross-over study design and furthermore our results would be strengthened by introducing a control group of students performing with bag valve mask ventilation (BVM). But within this study, the particular attention was turned to improvement in performance after a training programme, questioning whether or whether not this specific training concept might be sufficient. Besides, it is questionable which results BVM-ventilation would have shown in this setting. Recent studies of Noordergraaf et al. showed poor BVM ventilation of laypersons. In a clinical design, patients were ventilated by fireman first responders using a hand-held mask or an Oxylator. The working group could conclude that Oxylators perform significantly better (p < 0.0001) than the bag-valve device [16]. It seems therefore debatable whether inexperienced persons would be able to handle BVM sufficiently.

Our study has certain limitations that need to be addressed:

First, the students did not inflate cuffs by themselves. Hence, the students' performance may not clearly demonstrate lay users' ability to handle unknown airway devices and thus adequate ventilation correctly. Inflating the cuff of the device and connecting the bag-valve to the airway tool was done by an Instructor as well as the first ambu-bag™ compression resulting in a more comparable first ventilation. Reflecting real conditions, it might have been more precise to let the students themselves inflate the cuff. Our specific interest, however, was laid on the time frame between getting involved into the scenario and manually handling an unknown device. Thus, this procedure prevented faults or inappropriate handling with the connection between the valve-bag connector and the tube-side connector.

Second, gastric leakage, which is a potential risk caused by incomplete mask seal [5,17], was not precisely examined. We only registered audible sounds during first ambu-bag compression. Hence, this can at least give an idea of malposition leading to an increased risk of gastric air insufflation.

Third, we chose a tidal volume of < 150 ml (= dead space) as the threshold to define sufficient ventilation. The current ERC guidelines consider 400 ml with supplemental oxygen and even higher volumes without oxygen as sufficient [2].

It is debatable, though, if a manikin without any respiratory mechanics adequately reflects clinical conditions. Therefore we decided to at least filter cases where dead space ventilation occurred.

Besides, in retrospect, tidal volumes exceed ERC recommendations at every time point in the cases being identified as sufficient placement of the device.

In both groups, it could be shown that the tidal volume increased and cases of tidal volumes <150 ml decreased in the second evaluation and therefore, the placement of the device seems even more sufficient.

Nevertheless, even in the second evaluation, in 14 vs. 6 cases, tidal volume was <150 ml. Moreover, in both groups, two students were even unable to place the tool at all. It has to be considered that these were foreign students and that their poor performance can be explained by language problems.

In the other cases of tidal volume <150 ml further practical reasons might have played a role, like, for example, an unlubrified airway trainer.

Regarding the airway trainer, it must be considered that manikins representing the upper airway in studies can never replace human conditions sufficiently. The question whether these findings correlate with success rates in a real life situation has been unanswered. It is therefore debatable if the findings can be transferred into "real-life" clinical practice. Nonetheless, the intuitive use and the progress in performance could be shown clearly. Accordingly, these results might suggest that inaugurating laryngeal airway devices into BLS could be beneficial.

It is well known, that, in case of cardiopulmonary resuscitation, the early beginning is of paramount importance. For instance, integrating automated external defibrillators (AED) in BLS has already been recommended for several years. It has been shown previously that an AED can actually be used intuitively [18,19].

Concerning airway management and effective ventilation of a patient, intubating the trachea still brings out the "gold-standard" but should preferably be conducted without interrupting precordial compressions. Moreover, appropriate training and clinical experience are obligatory for the adequate performance of endotracheal intubation [2]. Alternatively, it has also been suggested to make use of laryngeal airway [20] devices for providing a secured airway and for reducing the risk of gastric regurgitation and tracheal aspiration [3-5]. The American Society of Anesthesiologists (ASA) implemented LMA as the first choice alternative in case of impossible or inadequate face-mask ventilation. In these guidelines, evidence from reviewed studies focussing on emergency laryngeal mask ventilation or the use of the ILM or LMA in case of difficult intubation suggest a successful airway access in 98% - 100%. [21].

Nevertheless, the ERC-Guidelines do not incorporate these devices into Basic Life Support. Our results may encourage the idea of implementing laryngeal airway devices into the BLS-Algorithm.

## Conclusion

Untrained laypersons are able to use different laryngeal airway devices sufficiently and might therefore arrange effective ventilation even without having any detailed technical information about the instrument. Taking into account that after minimal theoretical instruction and practical skill training of overall two hours the subjects improve significantly in their practical performance, these results underline the idea of implementing laryngeal airway devices into Basic Life Support. Hence, it is suggested that specific BLS-training programmes combined with airway management should be developed. Value has to be attached to general instructions and similarities. Describing specific details of the devices available seems not essential.

We believe that keeping instructions to lay people as simple as possible will additionly lead to more acceptance and motivation.

Concerning long time effects, it seems justified to suggest refreshment courses after six months as we could demonstrate retrograde results back to initial values for both devices.

## Abbreviations

LMA: laryngeal mask airway; BLS: Basic Life Support; ALS: Advanced Life Support; ERC: European Resuscitation Council; AED: Automated External Defibrillators; BVM: Bag Valve Mask; ILM: Intubation Laryngeal Mask.

## Competing interests

The authors declare that they have no competing interests.

## Authors' contributions

JB performed the statistical analysis and drafted the manuscript. MD, SB and GS carried out the acquisition of investigated results. MF participated in the design of the study and its coordination. RR conceived of the study and participated in its design and coordination and helped to draft the manuscript. All authors read and approved the final manuscript.

## Pre-publication history

The pre-publication history for this paper can be accessed here:


